# Porphyrin Aggregation Revisited: From the Four-Orbital Gouterman Model to an Eight-Orbital Framework in Porphin H‑Dimers

**DOI:** 10.1021/acs.jpca.6c02038

**Published:** 2026-07-03

**Authors:** Jannes Förster, Leo Cordsmeier, Vinícius Vaz da Cruz, Alexander Föhlisch

**Affiliations:** † Helmholtz-Zentrum Berlin für Materialien und Energie GmbH, Institute for Methods and Instrumentation for Synchrotron Radiation Research, Hahn-Meitner-Platz 1, 14109 Berlin, Germany; ‡ Humboldt-Universität zu Berlin, Unter den Linden 6, Berlin 10117, Germany; § Institut für Physik und Astronomie, Universität Potsdam, Karl-Liebknecht-Straße 24/25, Potsdam 14476, Germany

## Abstract

Porphyrin aggregation fundamentally alters photophysical properties, yet the underlying electronic structure reorganization remains poorly understood. Porphin H-type dimerization induces a characteristic blueshift of the B-band, explained within Kasha’s exciton model through dipole–dipole interactions, but this phenomenological picture does not resolve the frontier-orbital reorganization driving these changes. UV–vis spectroscopy and time-dependent density functional theory show spectral changes originate from systematic frontier-orbital splitting. The Gouterman four-orbital model extends to an eight-orbital framework in dimers through symmetric and antisymmetric combinations of the monomer HOMO–1, HOMO, LUMO, and LUMO+1. Configuration interaction within this expanded manifold governs B-band splitting, while natural transition orbital analysis shows Q-band excitations remain largely localized to single macrocycles. Nitrogen K-edge TDDFT reveals nearly unchanged core-level NEXAFS, confirming preserved local electronic structure despite valence reorganization. Aggregation thus extends Kasha’s exciton picture and the Gouterman model, providing an orbital-resolved foundation for excitonic effects in porphyrin assemblies.

## Graphical abstract

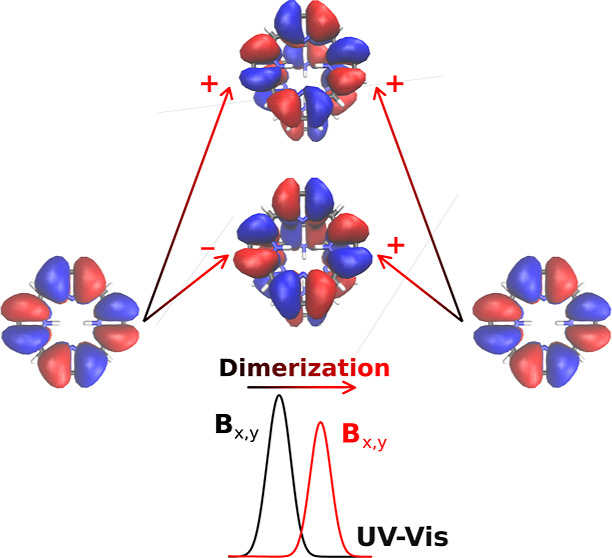

## Introduction

Porphyrins and their derivatives are ubiquitous in natural and synthetic light-harvesting systems,^1^ serving as chromophores in photosynthesis,^2^ photodynamic therapy,^3^ and dye-sensitized solar cells.^4^ Their characteristic optical properties arise from π–π* transitions between four frontier orbitals, the Gouterman orbitals,^5^ which generate the intense Soret (B) band around 3.2 eV and weaker Q bands between 2 and 2.5 eV.^6^ In biological and artificial environments, porphyrins frequently undergo spontaneous aggregation driven by π–π stacking interactions.^7,8^ This aggregation dramatically alters their photophysical properties: absorption bands shift in energy and intensity.^9^ Understanding and predicting these spectral changes is crucial for rational design of porphyrin-based light-harvesting systems, where controlled aggregation can be instrumental to maximizing the efficiency of a process.

**Table 1:** Electronic Transitions in the Porphin Monomer and H-Dimer Form from CAM-B3LYP TDDFT Calculations^a^

	monomer				dimer			
	E/eV	2*f_osc_	configuration	weight	E/eV	*f*_osc_	configuration	weight
*Q*_*x*_	2.22	0.004	5*b*_1u_ → 4*b*_2g_	0.50	2.21	0.002	see Supporting Information Figure 2a	
			2*a*_u_ → 4*b*_3g_	0.47	2.21	0.001		
*Q*_*y*_	2.39	0.014	5*b*_1u_ → 4*b*_3g_	0.45	2.37	0.003	see Supporting Information Figure 2b	
			2*a*_u_ → 4*b*_2g_	0.53	2.38	0.006		
*B*_*x*_	3.38	2.714	4*b*_1u_ → 4*b*_2g_	0.05	3.60	2.154	1*A*′ → 3*A*″	0.2
							1*A*″ → 4*A*′	0.32
			5*b*_1u_ → 4*b*_2g_	0.43			2*A*′ → 4*A*″	0.22
			2*a*_u_ → 4*b*_3g_	0.49			2*A*″ → 3*A*′	0.13
*B*_*y*_	3.43	3.009	5*b*_1u_ → 4*b*_3g_	0.53	3.57	2.157	1*A*′ → 4*A*′	0.31
							1*A*″ → 3*A*″	0.17
			2*a*_u_ → 4*b*_2g_	0.44			2*A*′ → 3*A*′	0.14
							2*A*″ → 4*A*″	0.26

a Excitation energies (E), oscillator strengths (*f*_osc_), dominant orbital configurations, and their weights are shown for the four lowest energy transitions. Monomer oscillator strengths are doubled to account for the two chromophores in the dimer. The blueshift of *B*_*x*_ and B_*y*_ transitions (0.22 and 0.14 eV) upon dimerization reflects H-type excitonic coupling, while Q-band transitions show a smaller redshift.

The spectral shifts observed in porphyrin aggregates have traditionally been interpreted using Kasha’s molecular exciton model,^9^ which treats individual chromophores as point dipoles interacting through their transition dipole moments. In this picture, the excitation energy splitting Δ*E* is determined by the geometric arrangement of dipoles: H-aggregates, where chromophores stack cofacially with parallel transition dipole moments, exhibit blueshifts as the symmetric (in-phase) combination is destabilized, while J-aggregates with head-to-tail geometries show redshifts due to stabilization of the lower excitonic state. This model has proven remarkably successful for rationalizing spectral changes in various molecular assemblies.^10,11^ While characterizing each monomer in an aggregate by its position and transition dipole moment provides qualitative insight into its spectroscopy, it does not offer information about the underlying orbital reorganization. At the separations typical of stacked porphyrin dimers (3 to 4 Å), molecular orbitals interact and split upon aggregation and overlap between orbitals is not negligible.^12^ A complete description requires understanding how the frontier orbitals themselves reorganize upon aggregation. First-principle approaches like time-dependent density functional theory (TDDFT) can therefore highlight the importance of these orbital-level reorganizations and are essential for a complete understanding of electronic transitions before and after aggregation.^13^

## Methods

A protocol by Khairutdinov and Serpone^14^ used to aggregate tetraphenylporphin was modified in order to get a spectral comparison of free base porphin in its aggregated and monomeric form. Porphin was purchased from Porphyrin Systems (Halstenbek, Germany) with a minimum purity of 98%. It was first dissolved in pure acetone to reach a concentration of 10 μ M. 50 mL of this solution was subsequently injected into 250 mL of water. Spectra were collected immediately after injection and measured again after 1 h to account for precipitation, though it yielded no significant spectral change. Measurements were carried out at room temperature on a Shimadzu UV-2700 with a path length of 1 cm. The baseline for the monomer measurement was a pure acetone sample, and a mixture of one part acetone to five parts water was used as a baseline for the aggregated sample.

All geometry optimizations and full TDDFT calculations were carried out with the Orca package,^15^ and visualizations were done with VMD: visual molecular dynamics.^16^ Molecular geometries were optimized using the CAM-B3LYP^17^ hybrid functional and ma-def2-QZVP^18^ basis set and def2/J^19^ auxiliary basis set. For the simulation of both the acetone and aqueous environment (resulting in the spectra shown in Figure 1), the conductor-like polarizable continuum model (CPCM^20^) has been used both in the geometry optimization and TDDFT calculations. Grimme’s D3­(BJ) dispersion correction^21^ was utilized in all calculations, which was found to be strictly necessary for accurate calculations of the dimer, together with a long-range corrected functional. The Tamm–Dancoff approximation^22^ was omitted, as excitation and deexcitation amplitudes are significantly coupled in the linear response. This is due to the strong configuration interaction present in porphin, so the inclusion of the B matrix thus has a strong stabilizing effect and lowers the overall transition energies by about 0.5 eV. For the nitrogen K-edge TDDFT, the excitation space was restricted to the nitrogen 1s core orbitals. The resulting stick spectra were broadened by convolution using a Voigt profile of 0.12 eV (Lorentzian fwhm) and 0.70 eV (Gaussian fwhm).

**Figure 1:**
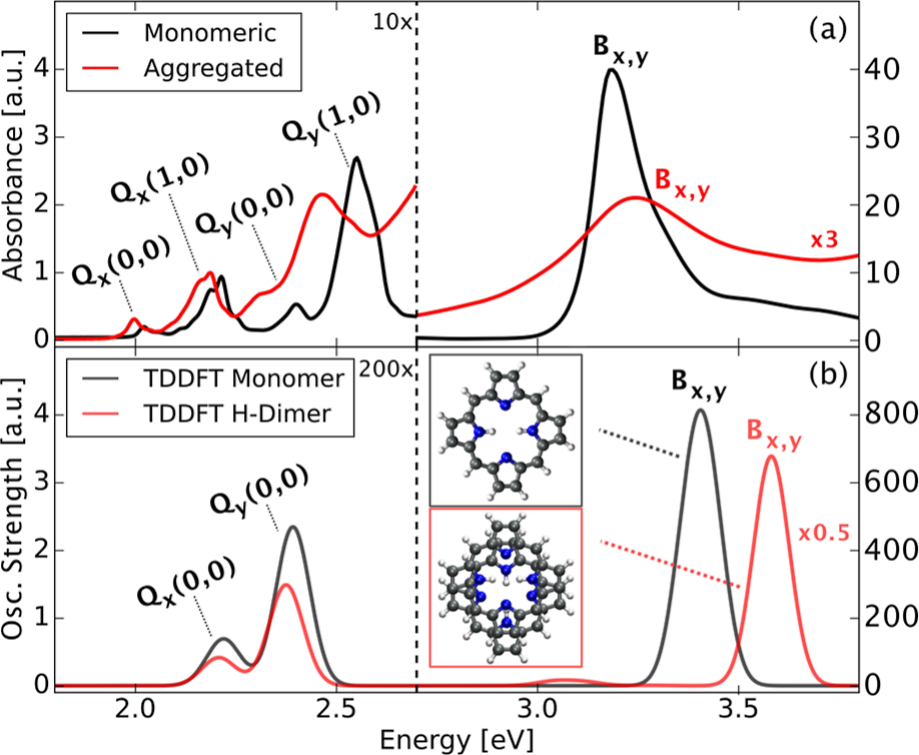
Comparison of UV–vis absorption spectra for porphin monomers and aggregates from the experiment and TDDFT calculations. (a) Experimental absorption spectra showing the B_*x*, *y*_ band region and Q-band transitions for monomeric (black) and aggregated (red) porphin. The aggregated spectrum exhibits a blueshifted and broadened Soret band and vibronically split Q-band peaks labeled Q_*x*_(0,0), Q_*x*_(1,0), Q_*y*_(0,0), and Q_*y*_(1,0), characteristic of D_2h_ porphins and their vibronic splitting. (b) TD-DFT calculated oscillator strengths using the CAM-B3LYP functional^17^ for porphin monomers (black) and H-dimers (red, halved for comparison), showing only electronic transitions without vibronic contributions. Inset molecular structures illustrate the cofacial stacking geometry of the H-dimer configuration. The calculated spectra qualitatively reproduce the blueshift and intensity redistribution of the Soret band.

## Results and Discussion

In this work, we investigate the electronic structure changes accompanying free-base porphin dimerization using UV–vis spectroscopy and TDDFT calculations. We demonstrate that the conventional four-orbital Gouterman model evolves naturally to an eight-orbital framework for the dimer: the frontier orbitals of each monomer combine to form eight dimer orbitals through symmetric and antisymmetric interactions, and transitions among these eight orbitals account for the observed spectral shifts. Previous TDDFT studies^23–25^ of porphin revealed that the conventional four orbital Gouterman model remains valid under modern quantum chemical methods. The characteristic absorption features can be attributed predominantly to transitions between the nearly degenerate HOMO–1, HOMO, LUMO, and LUMO+1 that are of the Gouterman form. Our approach reveals that aggregation preserves the essential orbital topology of the Gouterman model while introducing energetic splitting that directly manifests in the observed blueshift of the B band. Analysis of natural transition orbitals further shows that Q-band transitions remain largely localized on individual monomers, explaining their relative insensitivity to aggregation.

To obtain a UV–vis measurement of monomeric and dimerized porphin, a protocol by Khairutdinov and Serpone^14^ to aggregate tetraphenylporphin was modified. Porphin was first dissolved in pure acetone and 50 mL of this solution was injected into 250 mL of water. Upon injection, the color of the solution immediately changed from pink to yellow and the sample’s optical spectrum was measured. The results can be seen in Figure 1a alongside the porphin spectrum in pure acetone, where it is present in a predominantly monomeric form. The monomer spectrum is in good agreement with previously reported UV–Vis spectra of free-base porphin in organic solvents.^26,27^ A clear blueshift and broadening of the Soret band upon aggregation can be observed, accompanied by smaller broadening and redshift of the Q-band region. This behavior is characteristic of H-type excitonic coupling, in which the two adjacent porphin macrocycles align cofacially. The corresponding transition dipole moments are parallel and in-phase, arranged so that they cancel in the lowest exciton state according to Kasha’s exciton model.^9^ The strong broadening of peaks after injection likely occurs due to the mixture of monomeric and aggregated forms present in the solution.

To further rationalize the experimental spectra, TDDFT calculations were performed for several possible dimer geometries optimized at the CAM-B3LYP level of theory. The only dimer geometries found to converge were the face-to-face H-Dimer, which was found to be the most stable configuration by 0.17 eV compared to two less favored “T-shaped” aggregates,^28^ where the molecular planes form a right angle (see Supporting Information for details on the influence of intermolecular geometry on electronic spectra). The lowest energy “H-dimer” configuration is shown in Figure 1b, which will be the main focus of this work. The two molecules are offset orthogonal to their molecular planes by 3.17 Å. The macrocycles are also laterally displaced along their molecular planes by 1.98 Å, with one of them being rotated by 90°. This arrangement facilitates dipole–dipole interaction between the central N–H units of one ring with the nitrogen lone pairs of the adjacent ring. The nonpolar rest of the macrocycles are further stabilized by π–π stacking, driven by London dispersion.^7,8^

The computed absorption spectra for the optimized H-dimer reproduce the experimentally observed blueshift of the B-band upon aggregation, confirming that the H-dimer geometry utilized for the calculations accurately describes the observed experimental aggregation. TDDFT predicts a blueshift of 0.22/0.14 eV from 3.38/3.43 eV in the monomer to 3.60/3.57 eV in the dimer (see Table 1). In experiment, a shift of 0.04 eV is observed from 3.19 to 3.25 eV. This discrepancy can largely be attributed to the fact that the aggregated measurement is a combination of dimers, monomers, and other polyaggregate porphins present in equilibrium with each other. Thus, it is not representative of a pure H-dimer measurement. The much higher Q-Band intensity in the experiment is a result of intensity borrowing and Herzberg–Teller^29,30^ coupling, which is unaccounted for in the TDDFT calculations. We use the Franck–Condon approximation and neglect the nuclear degrees of freedom, as this greatly reduces computational complexity and still allows us to accurately predict B-band properties.

Orbital analysis of the computed monomer spectrum reproduces the established features of the Gouterman model. The *Q*_*x*_, *Q*_*y*_, *B*_*x*_, and *B*_*y*_ transitions are described by different linear combinations of the HOMO – 1, HOMO, LUMO, and LUMO + 1. The shapes of these orbitals and irreducible representations in the *D*_2h_ point group are shown in Figure 2.

**Figure 2:**
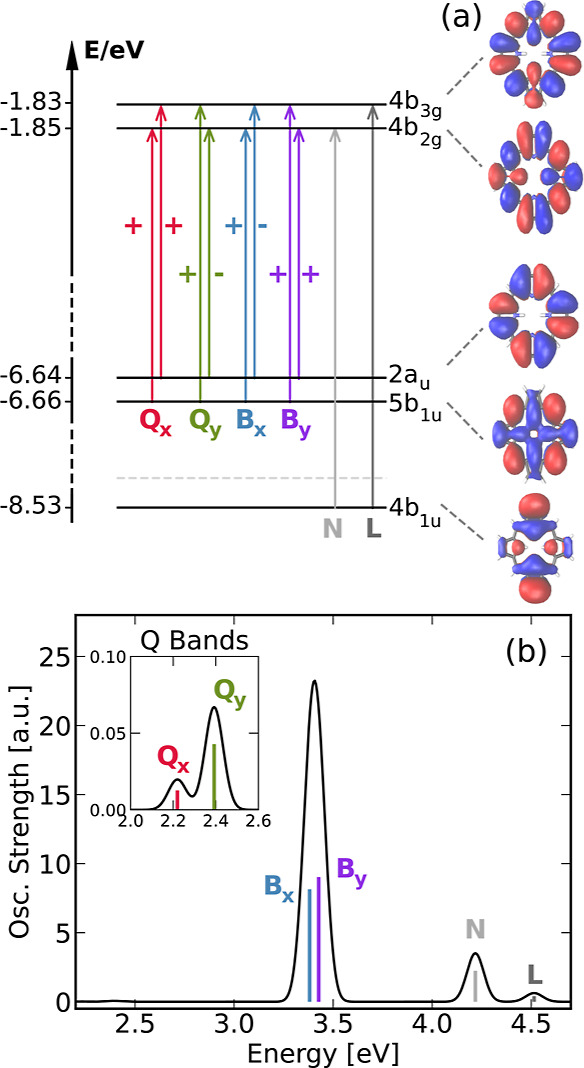
Electronic structure and excited-state transitions of the porphin monomer using TDDFT, confirming the Gouterman four-orbital model. (a) Molecular orbital diagram showing the two nearly degenerate HOMOs (2a_u_, 5b_1u_) around −6.65 eV and two nearly degenerate LUMOs (4b_2g_, 4b_3g_) around −1.84 eV, which comprise the Gouterman orbitals. Single transitions from the HOMO–3 4b_1u_ account for the higher energy N- and L-Bands. Arrows indicate electronic transitions generating the spectrum, with ± signs showing configuration interaction phases. (b) TDDFT-calculated oscillator strengths showing a weak, nearly forbidden Q-band at 2.3 eV and an intense B-band at 3.4 eV. The near-degeneracy of the Gouterman orbitals leads to strong configuration interaction, producing the characteristic split Q and B band patterns. N and L transitions appear at energies over 4 eV.

Upon dimerization, the system’s electronic structure reorganizes as the energetically close orbitals of each macrocycle perturb one another (Figure 3a). As a result, symmetric (same sign) and antisymmetric (opposite sign) combinations of their frontier orbitals emerge as solutions for the dimerized ground state. The point group of the dimerized system reduces from D_2h_ to C_s_, with A′ and A″ irreducible representations. However, orbital energy ordering and nodal patterns allow identification of monomer parentage. The four occupied dimer orbitals derive from symmetric and antisymmetric combinations of the two monomer HOMOs (a_u_) and HOMO – 1s (b_1u_). The four unoccupied orbitals arise from combinations between the LUMO (b_2g_) and LUMO + 1 (b_3g_) of each monomer.

**Figure 3:**
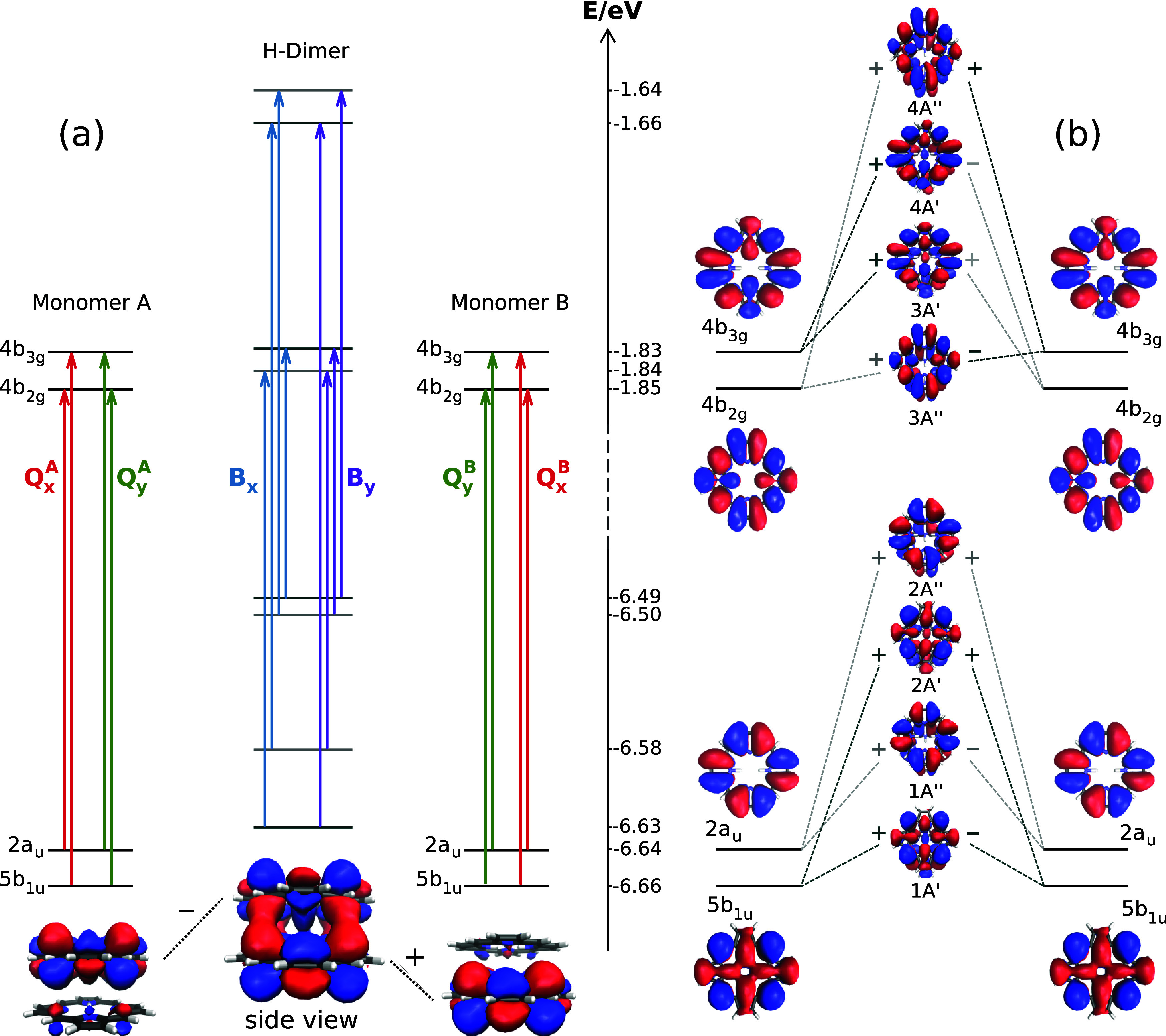
Electronic structure of the porphin H-dimer derived from exciton coupling of monomer Gouterman orbitals. (a) Excitonic coupling scheme showing how the Gouterman four-orbital transitions (*Q*_*x*_, *Q*_*y*_, *B*_*x*_, *B*_*y*_) of monomers A and B interact in the cofacial H-dimer geometry (shown in top and side views at bottom). Arrows indicate electronic transitions, with coupling between monomers leading to splitting of excitation energies. (b) Molecular orbital energy diagram illustrating the eight-orbital model for the H-dimer. The four Gouterman orbitals from each monomer combine through in-phase and out-of-phase interactions to produce eight dimer orbitals with C_s_ symmetry (A′ and A″ representations). Orbital visualizations show the spatial distribution and nodal patterns, with ± indicating bonding/antibonding combinations. The orbital splitting pattern determines the dimer’s excitonic states and spectroscopic properties.

In Figure 3b, the labels 1A′, 2A′, 1A″, 2A′′ (occupied) and 3A′, 4A′, 3A″, 4A′′ (unoccupied) denote the eight dimer orbitals arising from symmetric and antisymmetric combinations of the Gouterman orbitals, based on the irreducible representations of the dimer’s C_s_ point group. Single-electron transitions between this new set of eight orbitals comprise the electronic character of the shifted *B*_*x*_ and *B*_*y*_ transitions via configuration interaction. This extended orbital framework thus provides a consistent description of the excitonic splitting and orbital reorganization in porphin aggregation. Natural transition orbital analysis^31^ illustrates that in the Q-band region, the excitations remain localized on each individual monomer A and monomer B, with nearly independent *Q*_*x*_^A^, *Q*_*x*_^B^, *Q*_*y*_^A^, and *Q*_*y*_^B^ character, as seen in the side view in Figure 3a.

To probe whether aggregation affects the electronic structure beyond the frontier orbitals, we calculated N K-edge X-ray absorption spectra using CAM-B3LYP TDDFT, as core-level spectroscopy is known to be highly sensitive to the local electronic environment.^32^ Figure 4 shows the calculated N K-edge spectra for the monomer (black) and H-dimer (red), with dipole-allowed transitions from the N^Py^ (1b_2u_, 2a_g_) and N^PyH^ (1b_3u_) 1s core orbitals to the unoccupied Gouterman orbitals (4b_2g_, 4b_3g_) and the higher-lying 6b_1u_ orbital. Remarkably, the two spectra were nearly identical. This insensitivity stands in stark contrast to the pronounced changes observed in the valence UV–vis spectrum (Figure 1). The N K-edge transitions involve excitation of nitrogen 1s core electrons to the unoccupied Gouterman orbitals (4b_2g_, 4b_3g_) and the higher-lying 6b_1u_ orbital.

**Figure 4:**
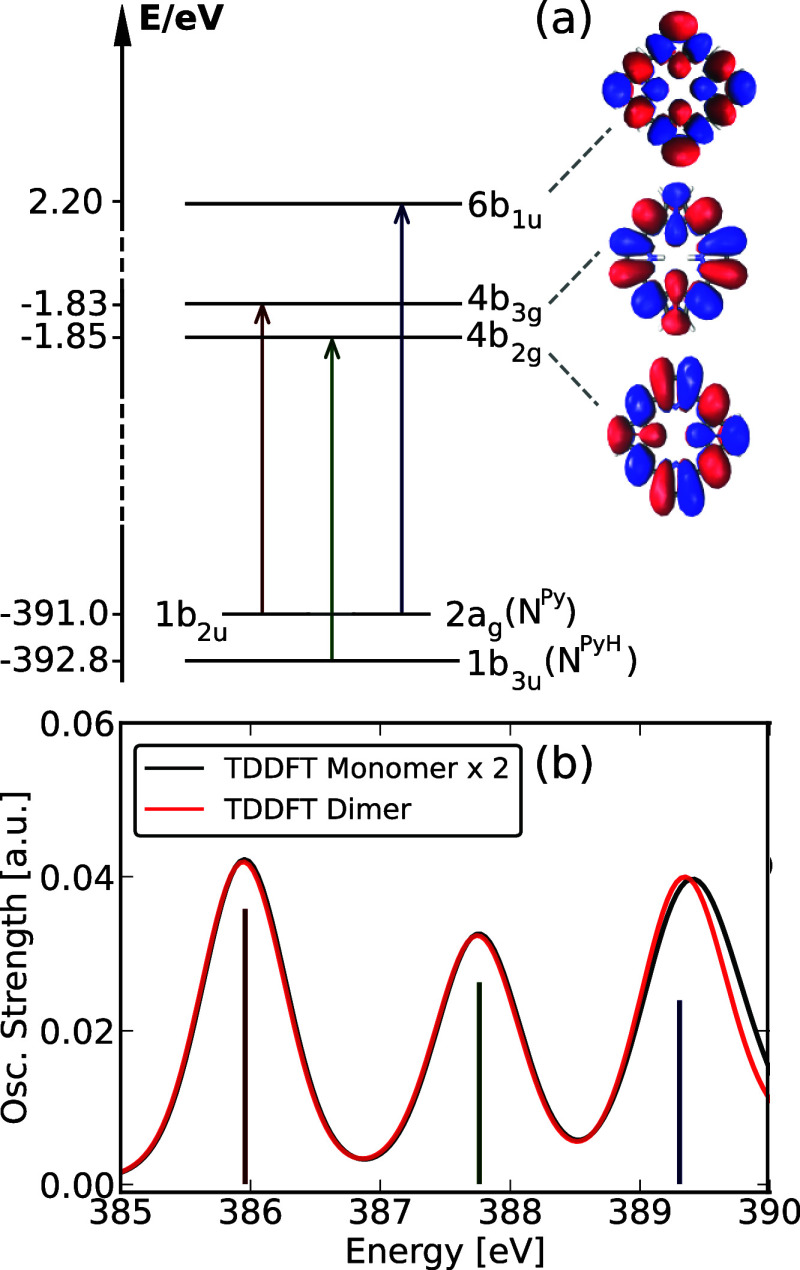
Calculated N K-edge X-ray absorption spectrum using CAM-B3LYP TDDFT. (a) Molecular core-excited states of the monomer, showing transitions from the N 1s core orbitals 1b_2u_, 2a_g_ (N^Py^, degenerate) and 1b_3u_ (N^PyH^) to the unoccupied Gouterman orbitals 4b_2g_, 4b_3g_ and the higher-energy 6b_1u_ orbital. The two distinct N 1s energies arise from the different chemical environments: the two pyrrolic nitrogens experience greater core-level shielding than the two pyridinic nitrogens. (b) Calculated TDDFT N K-edge spectra for monomer (black, scaled × 2) and H-dimer (red) show negligible differences in peak positions and intensities, indicating that aggregation does not alter the core-level shielding or the energy of transitions to unoccupied states, despite the changes in valence excitation energies.

The preservation of the monomer core-level spectrum indicates that the local electronic structure at the nitrogen site and the spatial distribution of the unoccupied orbitals remain essentially unperturbed by aggregation. This observation provides strong support for two key aspects of the eight-orbital model: first, it confirms that the orbital splitting upon dimerization is energetically small compared to the core-to-valence transition energies (around 390 eV), affecting only the relative energies of frontier orbitals while preserving their spatial character and local density. Second, it provides independent support that Q-band transitions remain localized on individual monomers, consistent with the natural transition orbital analysis (Figure 3a and Supporting Information).

## Conclusions

We have demonstrated that porphin dimerization induces a pronounced blueshift of the Soret band, consistent with H-type excitonic coupling. TDDFT calculations reproduce this shift and reveal that the conventional four orbital Gouterman model can be extended to an eight orbital analogue to capture the excitonic splitting in the dimer. Natural transition orbital analysis confirms that Q-band excitations remain localized on individual monomers, while B band transitions are the result of strong configuration interaction between the eight symmetric and antisymmetric combinations of frontier orbitals. N K-edge calculations independently validate this picture, showing preserved local electronic structure despite pronounced valence spectral shifts. Beyond the phenomenological description provided by Kasha’s exciton model, our analysis reveals how aggregation reorganizes the underlying frontier orbital structure, serving as a conceptual tool for interpreting spectral shifts in porphyrin assemblies. The eight-orbital framework should generalize to higher-order porphyrin aggregates and related macrocycles, with details like orbital parentage depending on specific geometry.

## Supplementary Material



## Notes

Molecular orbital configurations and weights for all transitions in the porphin monomer; TDDFT comparison of H-dimer, T-shaped aggregate, and trimer UV–vis spectra; natural transition orbitals of the porphin dimer Q-band; and dependency of TDDFT-computed electronic spectra on intermolecular geometry (PDF)
