# Supplementary material for: Porphyrin Aggregation Revisited: From the Four-Orbital Gouterman Model to an Eight-Orbital Framework in Porphin H‑Dimers
Source: J Phys Chem A. 2026 Jul 3;130(28):5380–5. doi: 10.1021/acs.jpca.6c02038 (PMC13383731; doi:10.1021/acs.jpca.6c02038)
Supplement: Supplementary file 1 [file jp6c02038_si_001.pdf]

[Supporting Information] Porphyrin Aggregation Revisited: From the Four-Orbital Gouterman Model to an Eight-Orbital Framework in Porphin H-Dimers

Jannes Förster,[*,†,‡] Leo Cordsmeier,[†,¶] Vinícius Vaz da Cruz,[*,†] and Alexander Föhlisch[*,†,¶]

†*Helmholtz-Zentrum Berlin für Materialien und Energie GmbH, Hahn-Meitner Platz 1, 14109 Berlin, Germany*

‡*Humboldt-Universität zu Berlin, Unter den Linden 6, 10117 Berlin, Germany*

¶*Universität Potsdam, Institut für Physik und Astronomie, Karl-Liebknecht-Straße 24/25, 14476 Potsdam, Germany*

E-mail: jannes.foerster@helmholtz-berlin.de; vinicius.vaz_da_cruz@helmholtz-berlin.de; alexander.foehlisch@helmholtz-berlin.de

Table S1: Energy, configuration and weights of the four lowest energy transitions in porphin using CAM-B3LYP level of theory

	E/eV	Configuration	Weight
Q_x	2.22	$5b_{1u} \rightarrow 4b_{2g}$	0.50
		$2a_u \rightarrow 4b_{3g}$	0.47
Q_y	2.39	$5b_{1u} \rightarrow 4b_{3g}$	0.45
		$2a_u \rightarrow 4b_{2g}$	0.53
B_x	3.38	$4b_{1u} \rightarrow 4b_{2g}$	0.05
		$5b_{1u} \rightarrow 4b_{2g}$	0.43
		$2a_u \rightarrow 4b_{3g}$	0.49
B_y	3.43	$5b_{1u} \rightarrow 4b_{3g}$	0.53
		$2a_u \rightarrow 4b_{2g}$	0.44
N	4.22	$4b_{1u} \rightarrow 4b_{3g}$	0.87
L	4.515	$4b_{1u} \rightarrow 4b_{2g}$	0.91

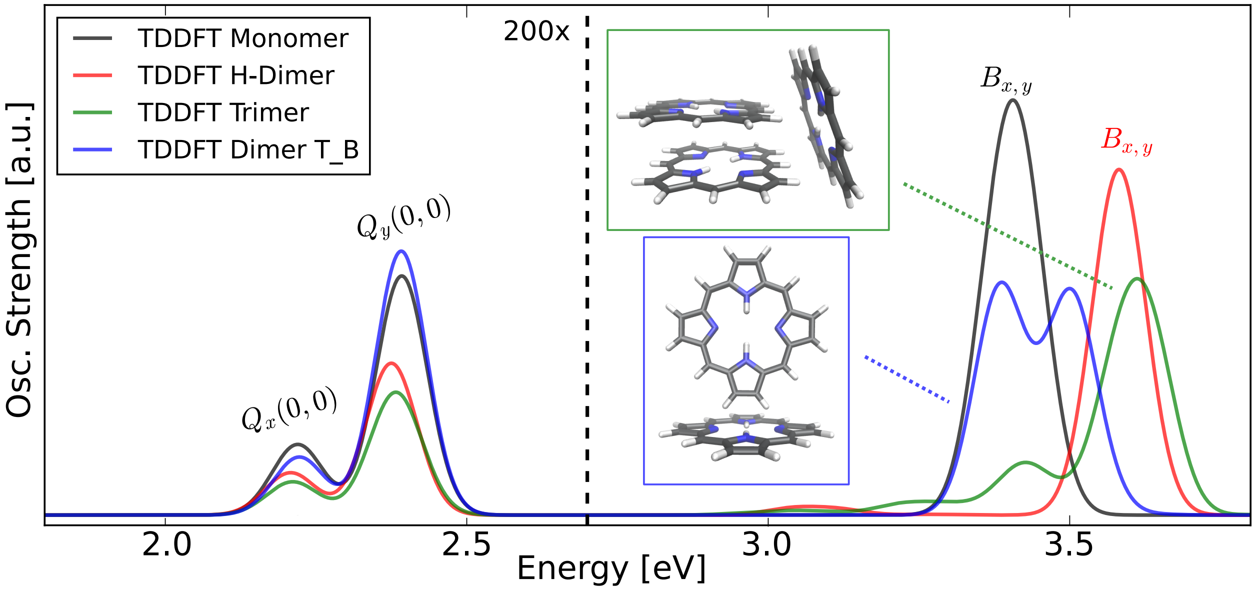

Figure S1: Comparison of UV-VIS absorption spectra for porphin monomers and aggregates from experiment and TDDFT calculations. Included is the energetically lower of two 'T-shaped' aggregates labelled T_B,[1] lying 0.17 eV above the H-Dimer. An optimized trimer geometry based on the H-aggregate is also shown, exemplifying how TDDFT predicts further aggregation also result in blueshifts.

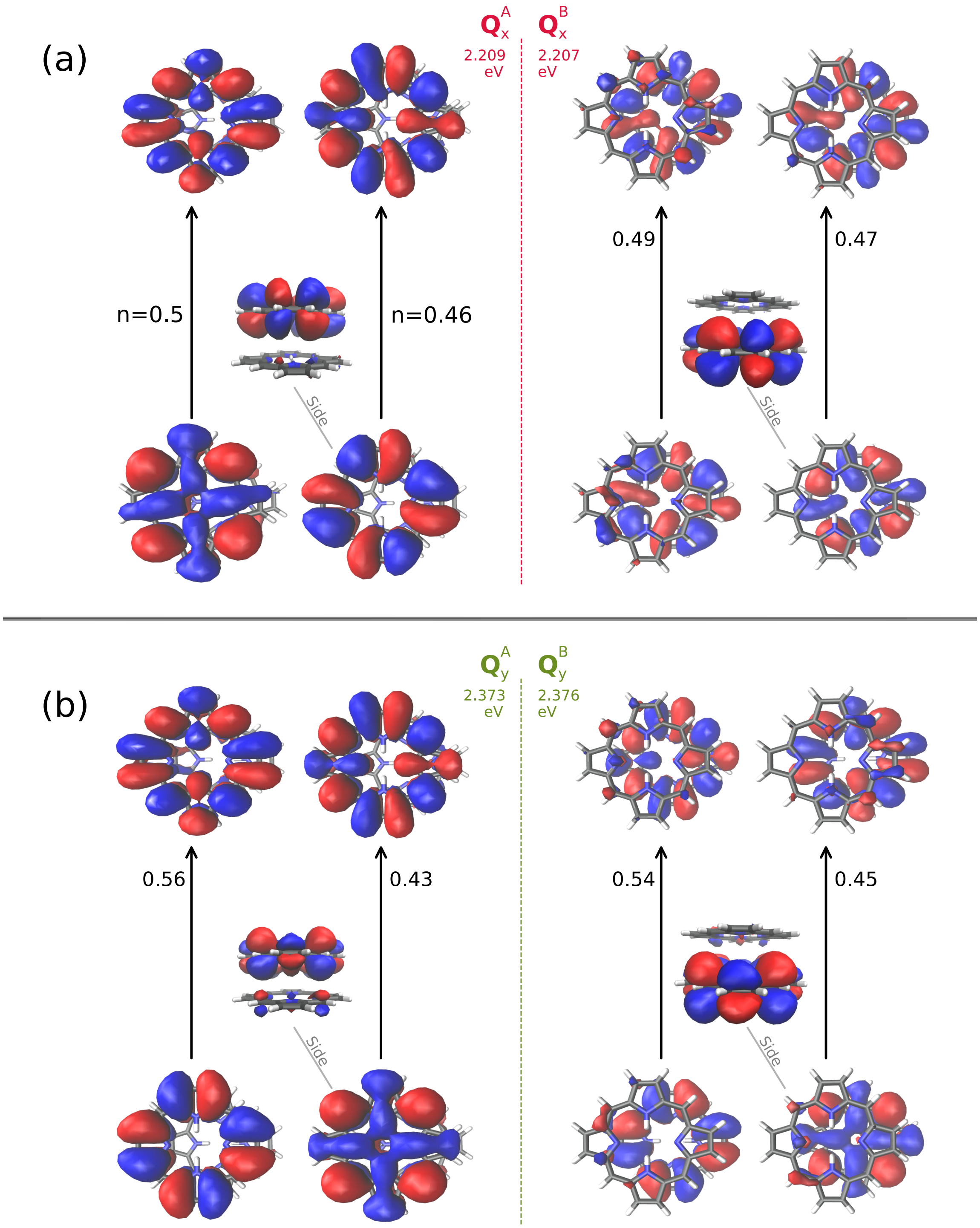

Figure S2: Natural transition orbitals of the porphin Dimer Q-Band. Nearly all of each transition happens localized to one monomer and the orbital geometries have a strong Gouterman character.

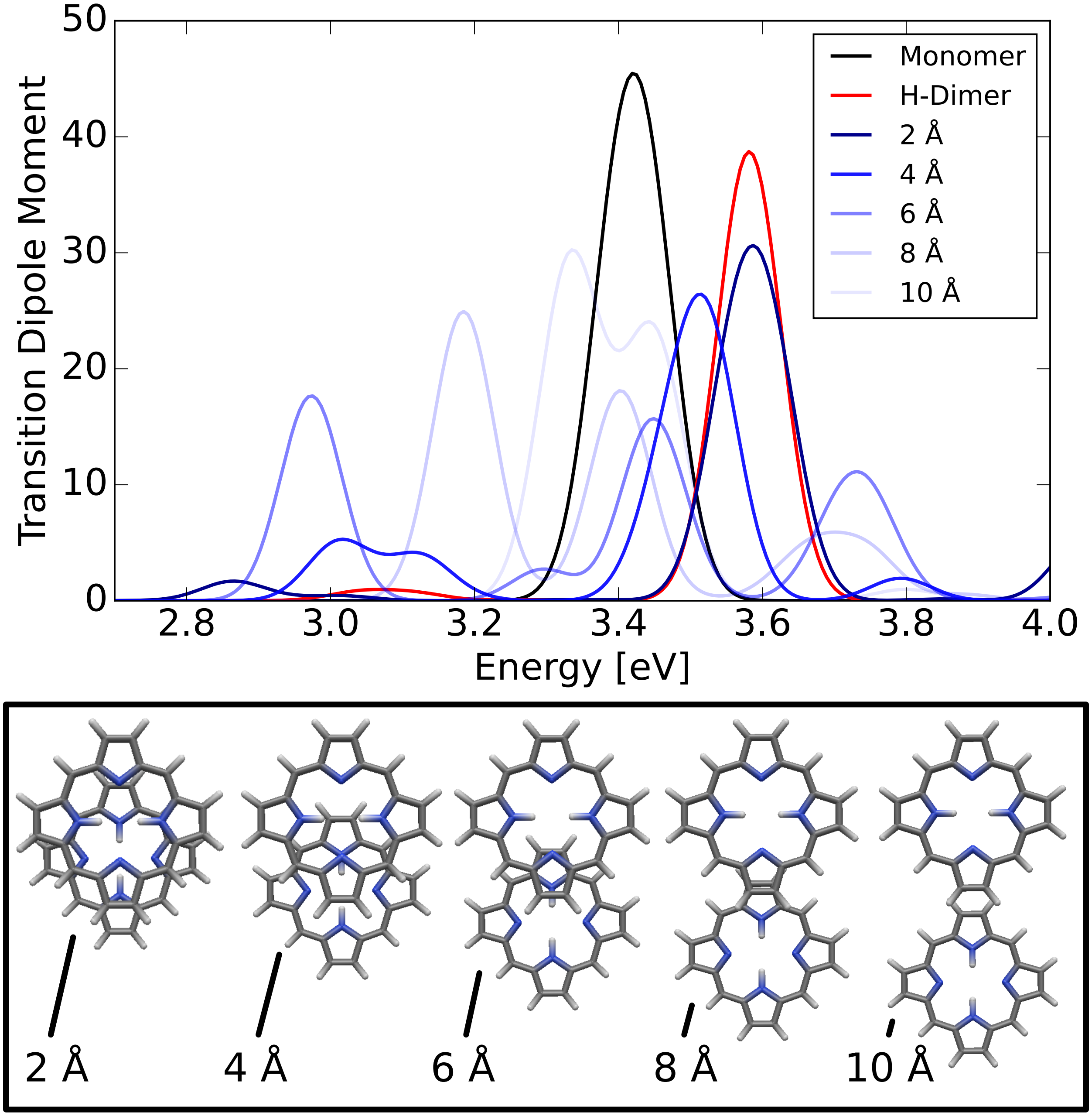

Figure S3: Dependency of TDDFT computed electronic spectra on intermolecular geometry. Interplanar separation is kept constant as the lateral displacement is incrementally increased in steps of 2 Å. The B-band at 3.6 eV in the H-dimer is shown to decrease and merge closer to the monomeric B-band position at 3.4 eV.

References

(1) Bergendahl, L. T.; Paterson, M. J. Excited states of porphyrin and porphycene aggregates: Computational insights. *Computational and Theoretical Chemistry* **2014**, *1040-1041*, 274–286, Excited states: From isolated molecules to complex environments.
